# Association between Frequency of Conversations and Suicidal Ideation among Medical Students during COVID-19 Pandemic in Japan

**DOI:** 10.3390/ijerph19116385

**Published:** 2022-05-24

**Authors:** Juri Yamazaki, Masashi Kizuki, Takeo Fujiwara

**Affiliations:** 1 Department of Global Health Promotion, Tokyo Medical and Dental University, Tokyo 113-8519, Japan; 180972ms@tmd.ac.jp; 2 Department of Tokyo Metropolitan Health Policy Advisement, Tokyo Medical and Dental University, Tokyo 113-8519, Japan; kizuki.tmhp@tmd.ac.jp

**Keywords:** COVID-19, social interaction, mental health, suicidal thought, students, Japan

## Abstract

To mitigate the spread of COVID-19, universities in Japan shifted from face-to-face to online classes, which might have reduced social interaction and increased psychiatric problems among students. A self-report questionnaire was administered to fourth-year medical students in Tokyo in May 2021, during the fourth wave of the COVID-19 outbreak, to examine the association between the frequency of conversations and suicidal thoughts. The questionnaire assessed the frequency of conversations and, using part of the Mini International Neuropsychiatric Interview, suicidal ideation. Of the 113 students, 98 (86.7%) responded, of whom 20 (20.4%) had suicidal ideation. Poisson regression analysis revealed that those with less than 1 conversation per week and no conversations at all had a significantly higher risk of suicidal ideation than those with 3 conversations per week or more, after adjusting for personality, family relationship, income level, living alone, number of friends, gender, and age. These results indicate that less frequent conversations increased the risk of suicidal ideation among medical students. Mental health support for students needs to be strengthened if universities suspend face-to-face classes during a pandemic.

## 1. Introduction

The coronavirus disease 2019 (COVID-19) pandemic and its countermeasures have caused people in the world to face social isolation and economic hardships [1,2,3], which could induce depression symptoms, worsening mental wellbeing [4]. At the beginning of the global pandemic, many experts were concerned that the rate of suicide mortality would increase [3]. Between July and October 2020, the monthly suicide mortality rate among children and adolescents increased by 49% compared to the previous year in Japan [5]. During the pandemic, children and adolescents might feel more psychological stress and have fewer protective factors against suicide such as social support.

In response to the outbreak, the Japanese government declared a state of emergency, which required targeted prefectures to take emergency measures. In Tokyo, people were asked to refrain from going out unnecessarily at all times [6]. In addition, public facilities larger than 1000 m^2^ were told to close, with events to limit their audience size to less than 5000 or 50% of the maximums, and restaurants were not to provide alcohol and were to close at 8 p.m. In the academic sector, most universities decided to shift to virtual classes and suspend all club activities. According to a survey by Japan’s Ministry of Education, Culture, Sports, Science, and Technology, 59.6% of university students attended lectures online all the time/almost all the time during the fall semester of 2020, as opposed to only 15% of students in higher education [7].

Under such a situation, university students rarely went out to meet their friends or lecturers, and their social interactions were greatly reduced [8]. Notably, in the survey, 53% of the university students who responded indicated that they felt lonely because they could not attend face-to-face lectures with their friends [7]. Therefore, it is reasonable to assume that students would have experienced social isolation [9,10,11]. Social isolation is assessed by the number of individuals whom one interacts with, the frequency of social interactions, the variety of relationships, and the degree of intimacy in the interactions [12]. Previous studies showed that social isolation was associated with suicidal ideation and suicidal attempt [13]. However, there were few interventions supported by adequate evidence to reduce social isolation [14]. Among the determinants of social isolation, a conversation is the most basic one; a systematic review showed cognitive behavioral therapy, which requires trained counselors and can reduce loneliness [15]. In a recent randomized control study, a phone call by a caller trained in empathetic conversational techniques reduced loneliness, depression, and anxiety [16]. In contrast, assessing the frequency of conversation, which is measurable and modifiable, does not need someone who is trained, that is, it is easily implemented, even online. Thus, we hypothesized that the frequency of conversation might be associated with suicidal ideation under the stressful COVID-19 pandemic. To investigate this hypothesis, we investigated the association between the frequency of conversation and suicidal thoughts in medical students during the COVID-19 pandemic in Japan.

## 2. Materials and Methods

### 2.1. Data

Data were collected by using a self-reported online questionnaire which was implemented in the class of fourth-year students at the medical school of Tokyo Medical and Dental University. The questionnaire was developed via Google form and the link to the survey site was distributed between 25 and 26 May 2021, which is under a state of emergency in Tokyo due to the fourth wave of the pandemic. Among 113 students, 98 students completed the questionnaire and were included in the present analysis (response rate, 86.7%).

### 2.2. Measurement

The frequency of conversation was measured by asking the question, “How often are you having conversations other than greetings (i.e., catching up on words)?” The respondents answered using the following options: “3 times per week or more”, “1 to 2 times per week”, “less than 1 time per week”, meaning 1 to 3 times per month, and “none”. The question did not define the format (chat, meeting, conference, lesson, etc.), mode (online, phone, face-to-face, etc.), minimum length, location of the conversation, or who the conversation was with.

Suicidal ideation was assessed by the first three questions taken from the modified Mini International Neuropsychiatric Interview (MINI) [17], which were “In the past month did you think that you would be better off dead or wish you were dead? (1 point)”, “In the past month did you want to harm yourself or to hurt or injure yourself? (2 points)” and “In the past month did you think about suicide? (6 points)”. We defined that a student had suicidal ideation if he/she answered yes to any of the three questions. We also calculated the strength of suicidal ideation as the total score for the three items (range: 0 to 9 points). A higher score indicated a higher risk of suicidal behavior [17].

We also collected information about covariates including gender (male and female), age at the timing of admission to the medical school (immediately after graduating from high school, after graduating from high school, and spending 1 or 2 years to pass the entrance exam, and after graduating from another university), personality traits, living alone (yes and no), family relationship (satisfactory and unsatisfactory), number of friends in the medical school, number of friends outside of the medical school, such as those from high school, and perceived level of personal income. Personality traits were assessed with the Big Five by using the Japanese version of the Ten-Item Personality Inventory (TIPI-J), and a score was calculated for each trait of extraversion, agreeableness, neuroticism, conscientiousness, and openness [18]. These covariates were selected based on their known association with either frequency of conversation or suicidal thoughts. For example, the risk of suicidal thoughts is higher among girls than boys [19]. Among the Big 5 personality traits, neuroticism elevated the levels of depression and was associated with a higher risk of suicide attempts while extraversion and conscientiousness were associated with a lower risk of depression [20]. Problems in family relationships, such as less caring and overprotective mothers [21] and child maltreatment [22], were associated with suicidal behavior, while satisfaction in family relationships reduced the risk of depressive feelings and suicidal ideation among adolescents [23,24]. Low perceived household economic status was associated with a higher prevalence of depressive symptoms and suicidal ideation under the COVID-19 pandemic [25]. Living alone was reported as one of the sociodemographic risk factors for suicide [26]. High school students whose peers were more isolated and had fewer friendship ties had a higher risk of suicidal ideation [27].

### 2.3. Statistical Analysis

We used both a binary variable (yes and no) and a continuous variable (total score) for suicidal ideation to improve the robustness of the findings. Poisson regression analyses were used to examine whether the risk (prevalence) of having suicidal ideation was associated with the frequency of conversation by using the binary variable for suicidal ideation as the outcome variable. Following the recommendation by Barros and Hirakata [28], Poisson regression which estimates the prevalence ratio was used because suicide ideation was 20.4% in the present sample and was not considered a rare event. As the cut-off for rare events, we applied 10% based on Thompson and Kriebe, who recommended prevalence ratios rather than prevalence odds ratios in cross-sectional data when the rare disease assumption was not met [29]. Linear regression was used to examine whether the strengths of suicidal ideation, as evaluated by the total score for three items related to suicidal ideation, were associated with the frequency of conversation.

Regarding the frequency of conversation, we defined the reference category for the variable as 3 times per week or more in the present analyses. To examine the influence of categorization on the results, we also grouped “less than 1 time per week” and “none” in a single category and repeated the analysis (results are presented in the supplementary material).

To control for potential confounders of the association between frequency of conversation and suicidal thought, we adjusted results for gender and age, evaluated as the timing of admission to the medical school, in model 1, and for personality, family relationship, perceived level of personal income, living alone, and number of friends, in addition to gender and age, in model 2.

We examined the presence of interaction between the frequency of conversation and the number of friends by including the interaction terms in the Poisson regression models and assessed the statistical significance of the interaction term. Since the interaction terms were not statistically significant for interaction terms between frequency of conversation and number of friends both outside and in medical school in both Poisson and linear regression analyses, we did not include them in the final model.

## 3. Results

A total of 20 participants were judged to have suicidal ideation. Characteristics of students by whether they had suicidal ideation or not are shown in Table 1. In Table 1, results for categorical variables are presented as frequency and percentages, and those for continuous variables as mean and standard deviation. There was no missing value in the data, therefore percentages, mean, and standard deviation were calculated among all subjects (N = 98).

The results of Poisson regression are shown in Table 2. There was a significant negative association between the frequency of conversation and the prevalence of suicidal ideation. Compared with those who had conversations three times per week or more, those with less than one time per week and none showed a higher risk of suicidal ideation, with a prevalence ratio (PR) of 6.54, and a 95% confidence interval (CI) of 1.18 to 36.21; and PR 9.30, 95% CI: 1.41 to 61.06 in the fully adjusted model, respectively. There is no significant difference between those with one to two times per week of conversation and those with conversations three times per week or more.

Similar results were obtained in linear regression (Table 3). There was a significant negative association between the frequency of conversation and the strength of suicidal ideation. Compared with those with three times per week or more conversation, those with less than one time per week conversation and none were more likely to have a higher degree of suicidal ideation, which have regression coefficient of 4.30 (95%CI: 1.68 to 6.92) and 3.36 (95%CI: 0.96 to 5.75) in the fully adjusted model, respectively. There was no significant difference in the strengths of suicidal ideation between those with one to two times per week of conversation and those with three times per week or more of conversation.

We repeated the analyses after combining the frequency of conversation of “less than 1” and “none” into a single category and confirmed that the results were qualitatively similar (Supplementary Tables S1 and S2).

## 4. Discussion

Among fourth-year medical students, a higher frequency of conversations was associated with lower prevalence and lower strengths of suicidal ideation. Specifically, compared with medical students with conversations three times per week or more, students with conversations less than one time per week showed higher risk and a higher degree of suicidal ideation. There is no significant difference between students with conversations one to two times per week and those with conversations three times per week or more.

Previous studies had shown that social isolation was associated with suicidal thoughts and suicidal attempts [13]. Socially isolated female adolescents in the United States, i.e., no friendship nominations from others, showed a higher risk for suicidal ideation [30]. Adolescents in China with no friends showed a higher risk for suicidal ideation and suicide attempts [31]. These studies did not uncover the mechanism of how social isolation leads to suicidal ideation among adolescents. The current study added to the literature that more frequent conversation is needed to prevent suicide among university students during the COVID-19 pandemic, independent of the number of friends.

Suicidal ideation is one of the severe symptoms of depression. A meta-analysis of longitudinal studies showed that those with depression showed a higher risk of suicidal ideation, attempt, and death [32]. 

Cognitive impairment is often observed in patients with depression. It is estimated that two-thirds of the depressive patients have occurred cognitive impairment [33]. There is a meta-analysis that shows that cognitive impairment represents depression that persists beyond symptoms of low mood, which implies that cognitive disfunction would be a valuable target for intervention before the patients become depressed [34].

To improve and maintain cognitive function, it is important to build a social network. For elderly people, both structural and functional poor social relationships predicted cognitive decline [35]. Moreover, lower frequency of conversation might affect future cognitive impairment. In a longitudinal study, with healthy elderly people, conducted in Wales, social isolation is associated with not only current cognitive function but also cognitive reserve [36]. Similar to these studies above, the association between social relationships and the cognitive function of elderly people is widely discussed. On the other hand, this association among younger populations is not so much focused on. If our study shows that social interaction, which is the frequency of conversation, leads to cognitive decline similar to the elderly population, and suicidal thoughts appear as its consequence, to understand how poor social interaction develops suicidal thought, further research that examines the association between social interaction and cognitive function among adolescents is needed.

We adjusted results for gender, age, personality, family relationship, perceived level of personal income, living alone, and number of friends in the regression analyses. The prevalence ratio (in Table 2), as well as regression coefficients (in Table 3) for the frequency of conversations, increased after the adjustment (Model 2 vs. Model 1), suggesting the presence of negative confounding by these factors. In the Poisson regression analysis, neuroticism and unsatisfactory family relationship showed a tendency to be associated with a higher prevalence of suicidal ideation. In the linear regression analysis, neuroticism showed a tendency to be associated with conscientiousness, and unsatisfactory family relationship showed significant association with a higher score for suicidal ideation. Neuroticism has been known as a risk factor for depression [20] and the above findings match previous studies. However, conscientiousness, which is usually associated with a lower level of depression [20], was associated with a higher risk of suicidal ideation in our population. Previous studies showed that a variety of family problems are associated with suicidal behaviors, and the present results are consistent with previous literature [21,22,37].

Several limitations need to be addressed. First, our sample did not represent all university students and the findings might not be generalized to all university students in Japan. University students in general went to campus an average of 2.6 days per week in the 2021 spring semester, according to the survey conducted by the National Federation of University Co-operative Associations [38]. Moreover, in the same survey, 34.7% of the students of universities in Tokyo answered that they did not go to campus at all and 25.3% of them answered that they went to campus once a week [38]. As medical students received the practicum, such as anatomy, which was implemented onsite, it was assumed that medical students had more opportunities to go to the campus, and university students in other fields might have received lectures only more and experienced social isolation more than the medical students under the COVID-19 pandemic. Medical students were not easily influenced by wrong information on COVID-19 from social media [39]. Further research investigating the association between the frequency of conversations and suicidal ideation among other universities is needed. Second, according to a survey by Japan’s Ministry of Education, Culture, Sports, Science, and Technology, first-year university students were more likely to suffer from friendship problems compared to those who were upper graders [7]. It is assumed that the prevalence of suicidal ideation among first-year medical students is higher than the one in the current study. Third, there might be errors in measurement of the frequency of conversations due to reliance on self-report. However, both objective conditions and subjective feelings were reported to be associated with suicidal outcomes, especially in a suicide attempt and suicidal ideation [13]. It is expected that a similar association would have been observed if we had used objective measurement of the frequency of conversation. In the same way, there might be an error in the assessment of the prevalence of suicidal ideation among medical students. However, medical students are supposed to respond to suicidal ideation questionnaires more accurately than other university students, because they would have high health literacy in general. The assessment of confounders also may not be accurate, which would cause residual confounding to estimate the true association between the frequency of conversations and suicidal ideation. Economic difficulties are widely known to be a risk factor for suicide [2], which means that there is a need to adjust for it. In the current study, we used perceived income level to assess economic status because 26.5% of the participants answered that they did not know their parents’ annual income in our survey. However, the perceived income level does not accurately reflect the current economic status. Therefore, this study might underestimate the effect of economic difficulties during the COVID-19 pandemic on suicidal ideations.

## 5. Conclusions

Having conversations less than one time per week increases the risk of suicidal ideation among university students, independent of the number of friends. Universities need to consider the opportunities for interactions between students, especially in online lectures.

## Figures and Tables

**Table 1:** Descriptive statistics of characteristics of students.

		Total (N = 98)	Not Having Suicidal Ideation (N = 78)	Having Suicidal Ideation (N = 20)
Categorical Variables		n (%)	n (%)	n (%)
Gender	Male	62 (63.3)	47 (60.2)	15 (75.0)
	Female	36 (36.7)	31 (39.7)	5 (25.0)
Time of admission to medical school	Immediately after graduating from high school	66 (67.3)	53 (67.9)	13 (65.0)
	Taking 1 or 2 years after graduating from high school to pass the entrance exam	18 (18.4)	14 (17.9)	4 (20.0)
	After graduating from another university	14 (14.3)	11 (14.1)	3 (15.0)
Living alone	No	70 (71.4)	56 (71.8)	14 (70.0)
	Yes	28 (28.6)	22 (28.2)	6 (30.0)
Frequency of conversations	3 times per week or more	78 (79.6)	65 (83.3)	13 (65.0)
	1 to 2 times per week	11 (11.2)	10 (12.3)	1 (5.0)
	Less than 1 time per week	4 (4.1)	1 (1.3)	3 (15.0)
	None	5 (5.1)	2 (2.6)	3 (15.0)
**Continuous variables**		**Mean (SD)**	**Mean (SD)**	**Mean (SD)**
Personality traits	Extraversion	7.67 (0.28)	7.58 (0.31)	8.00 (0.66)
	Agreeableness	9.86 (0.20)	10.00 (0.22)	9.35 (0.49)
	Neuroticism	8.10 (0.28)	7.96 (0.32)	8.65 (0.56)
	Conscientiousness	6.53 (0.24)	6.48 (0.26)	6.70 (0.58)
	Openness	7.73 (0.26)	7.58 (0.28)	8.30 (0.64)
Number of friends	Friends in medical school	6.72 (0.40)	6.90 (0.46)	6.05 (0.88)
	Friends outside of medical school	19.60 (1.02)	19.46 (1.16)	20.15 (2.14)
Perceived level of personal income		3.66 (0.08)	3.69 (0.09)	3.55 (0.23)

SD: standard deviation. There was no missing value in the data and the mean was calculated from all respondents (N = 98).

**Table 2:** Results of Poisson regression analysis about the prevalence of suicidal thoughts.

		Model 1			Model 2		
		PR	95% CI	*p*-Value	PR	95% CI	*p*-Value
Frequency of conversations	3 times per week or more	ref.			ref.		
	1 to 2 times per week	0.52	0.06, 4.02	0.53	0.78	0.08, 6.92	0.82
	Less than 1 time per week	4.19	1.11, 15.81	0.03	6.54	1.18, 36.21	0.02
	None	3.40	0.88, 13.06	0.07	9.30	1.41, 61.06	0.02
Gender	Male (ref.: female)	1.27	0.42, 3.83	0.66	1.44	0.43, 4.77	0.54
Time of admission to medical school	Immediately after graduating from high school	ref.			ref.		
	Taking 1 or 2 years after graduating from high school to pass the entrance exam	0.85	0.27, 2.70	0.79	1.13	0.29, 4.32	0.85
	After graduating from another university	0.87	0.24, 3.12	0.83	0.99	0.21, 4.58	0.99
Personality traits	Extraversion				1.14	0.94, 1.39	0.17
	Agreeableness				0.96	0.73, 1.26	0.78
	Neuroticism				1.22	0.97, 1.53	0.07
	Conscientiousness				1.05	0.85, 1.29	0.61
	Openness				0.99	0.79, 1.24	0.95
Living alone	Yes (ref.: No)				0.75	0.20, 2.76	0.66
Unsatisfactory family relationship					1.60	0.93, 2.97	0.08
Number of friends in medical school					0.93	0.80, 1.07	0.33
Number of friends outside of medical school					1.02	0.97, 1.09	0.33
Perceived level of personal income					1.11	0.57, 2.16	0.75

PR: prevalence ratio, CI: confidence interval, ref.: reference. Results were adjusted for gender and age (at the time of admission to medical school) in model 1, and for personality traits, living alone, family relationship, number of friends, and perceived level of personal income, in addition to gender and age, in model 2.

**Table 3:** Results of linear regression analysis about the total score for suicidal thoughts.

		Model 1			Model 2		
		Coef.	95% CI	*p*-Value	Coef.	95% CI	*p*-Value
Frequency of conversations	3 times per week or more	ref.			ref.		
	1 to 2 times per week	0.01	−1.52, 1.55	0.53	0.12	−1.53, 1.78	0.88
	Less than 1 time per week	4.46	1.99, 6.94	<0.01	4.30	1.68, 6.92	<0.01
	None	2.88	0.64, 5.13	0.01	3.36	0.96, 5.75	<0.01
Gender	Male (ref.: Female)	0.30	−0.72, 1.33	0.56	0.28	−0.80, 1.36	0.61
Time of admission to medical school	Immediately after graduating from high school	ref.			ref.		
	Taking 1 or 2 years after graduating from high school to pass the entrance exam	−0.55	−1.82, 0.72	0.39	−0.27	−1.60, 1.04	0.67
	After graduating from another university	−0.19	−1.60, 1.20	0.78	−0.01	−1.49, 1.46	0.98
Personality traits	Extraversion				−0.03	−0.23, 0.16	0.73
	Agreeableness				−0.05	−0.32, 0.21	0.68
	Neuroticism				0.16	−0.02, 0.35	0.09
	Conscientiousness				0.24	0.02, 0.45	0.03
	Openness				0.04	−0.18, 0.27	0.69
Living alone	Yes (ref.: No)				−0.21	−0.23, 0.16	0.73
Unsatisfactory family relationship					0.53	0.01, 1.06	0.04
Number of friends in medical school					0.01	−0.11, 0.14	0.87
Number of friends outside of medical school					0.01	−0.03, 0.07	0.50
Perceived level of personal income					0.09	−0.57, 0.76	0.77

Coef.: partial regression coefficient, CI: confidence interval, ref.: reference. Results were adjusted for gender and age (at the time of admission to medical school) in model 1, and for personality traits, living alone, family relationship, number of friends, and perceived level of personal income, in addition to gender and age, in model 2.

## Supplementary Materials

The following supporting information can be downloaded at: https://www.mdpi.com/article/10.3390/ijerph19116385/s1, Table S1: Results of Poisson regression analysis about the prevalence of suicidal thoughts; Table S2: Results of linear regression analysis about total score for suicidal thoughts.
