# Supplementary material for: Association between Frequency of Conversations and Suicidal Ideation among Medical Students during COVID-19 Pandemic in Japan
Source: Int J Environ Res Public Health. 2022 May 24;19(11):6385. doi: 10.3390/ijerph19116385 (PMC9180649; doi:10.3390/ijerph19116385)
Supplement: Supplementary file 1 [file ijerph-19-06385-s001.zip › ijerph-1701037-supplementary.pdf]

Supplementary Table S1. Results of Poisson regression analysis about the prevalence of suicidal thoughts.

		Model 1			Model 2		
		**PR**	**95% CI**	**p-value**	**PR**	**95% CI**	**p-value**
Frequency of conversations	3 times per week or more	ref.			ref.		
	1 to 2 times per week	0.52	0.06, 4.02	0.53	0.77	0.08, 6.79	0.82
	Less than 1 time per week or none	3.76	1.29, 10.94	0.01	7.6	1.84, 31.42	<0.01
Gender	Male (ref.: Female)	1.27	0.42, 3.83	0.66	1.43	0.43, 4.73	0.55
Time of admission to medical school	Immediately after graduating from high school	ref.			ref.		
	Taking 1 or 2 years after graduating from high school to pass the entrance exam	0.84	0.26, 2.66	0.77	1.18	0.32, 4.34	0.8
	After graduating from another university	0.87	0.24, 3.13	0.84	0.99	0.21, 4.50	0.99
Personality traits	Extraversion				1.14	0.94, 1.39	0.16
	Agreeableness				0.96	0.73, 1.26	0.79
	Neuroticism				1.22	0.97, 1.52	0.07
	Conscientiousness				1.05	0.85, 1.28	0.63
	Openness				0.98	0.79, 1.22	0.89
Living alone	Yes (ref.: No)				0.82	0.25, 2.62	0.74
Unsatisfactory family relationship					1.61	0.94, 2.75	0.07
Number of friends in medical school					0.93	0.81, 1.07	0.35
Number of friends outside of medical school					1.02	0.97, 1.08	0.36
Perceived level of personal income					1.17	0.65, 2.10	0.59

PR: prevalence ratio, CI: confidence interval, ref.: reference.

Regarding the frequency of conversations, "less than 1" and "none" were combined into a single category. Results were adjusted for gender and age (as the time of admission to medical school) in model 1, and for personality traits, living alone, family relationship, number of friends, and perceived level of personal income in addition to gender, and age in model 2.

Supplementary Table S2. Results of linear regression analysis about total score for suicidal thoughts.

		Model 1			Model 2		
		**Coef.**	**95% CI**	**p-value**	**Coef.**	**95% CI**	**p-value**
Frequency of conversations	3 times per week or more	ref.			ref.		
	1 to 2 times per week	0.01	-1.52, 1.55	0.53	0.17	-1.47, 1.82	0.83
	Less than 1 time per week or none	3.59	1.85, 5.33	<0.01	3.75	1.91, 5.58	<0.01
Gender	Male (ref.: Female)	0.30	-0.72, 1.33	0.56	0.30	-0.77, 1,38	0.58
Time of admission to medical school	Immediately after graduating from high school	ref.			ref.		
	Taking 1 or 2 years after graduating from high school to pass the entrance exam	-0.58	-1.86, 0.68	0.36	-0.35	-1.66, 0.95	0.59
	After graduating from another university	-0.19	-1.59, 1.21	0.78	0.03	-1.42, 1.50	0.95
Personality traits	Extraversion				-0.02	-0.23, 0.16	0.78
	Agreeableness				-0.06	-0.32, 0.21	0.65
	Neuroticism				0.16	-0.02, 0.35	0.10
	Conscientiousness				0.24	0.02, 0.45	0.02
	Openness				0.03	-0.18, 0.27	0.75
Living alone	Yes (ref.: No)				-0.28	-1.50, 0.92	0.63
Unsatisfactory family relationship					0.54	0.02, 1.05	0.04
Number of friends in medical school					-0.06	-0.55, 0.41	0.77
Number of friends outside of medical school					0.05	-0.14, 0.25	0.59
Perceived level of personal income					0.06	-0.57, 0.69	0.85

Coef.: partial regression coefficient, CI: confidence interval, ref.: reference.

Regarding the frequency of conversations, "less than 1" and "none" were combined into a single category. Results were adjusted for gender and age (as the time of admission to medical school) in model 1, and for personality traits, living alone, family relationship, number of friends, and perceived level of personal income in addition to gender, and age in model 2.
